# Oxytocin, Epigenetic Aging, and the Social Regulation of Health: A Lifecourse Perspective on the Maejima et al. Findings

**DOI:** 10.1111/acel.70363

**Published:** 2026-01-30

**Authors:** Kerstin Uvnäs‐Moberg, Mechthild M. Gross, Jean Calleja‐Agius, Jonathan D. Turner

**Affiliations:** ^1^ Department of Animal Environment and Health, Section of Anthrozoology and Applied Ethology Swedish University of Agricultural Sciences Skara Sweden; ^2^ Midwifery Research and Education Unit Hannover Medical School Hannover Germany; ^3^ Department of Anatomy, Faculty of Medicine and Surgery University of Malta Msida Malta; ^4^ Immune Endocrine Epigenetics Research Group, Department of Infection and Immunity Luxembourg Institute of Health Esch sur Alzette Luxembourg

**Keywords:** DNA methylation, early life programming, epigenetic aging, HPA axis, lifecourse epidemiology, noradrenergic/sympathetic nervous system, oxytocin, personalized epigenetic medicine, social connection, TET enzymes

## Abstract

The elegant work by Maejima et al. recently published in *Aging Cell* reveals a previously unrecognized mechanism linking age‐related oxytocin (OXT) decline to epigenetic remodeling, mitochondrial dysfunction, and systemic inflammation (Maejima et al. 2025). Beyond documenting this relationship, the authors demonstrate its remarkable reversibility through nasal OXT administration. These findings provide the first molecular evidence supporting what has long been proposed: that the OXT system functions as a fundamental long‐term regulator of health across the entire lifespan, from early development through aging (Moberg 2024, 2003; Uvnas‐Moberg 1998). The current work now gives a tantalizing glimpse into the epigenetic mechanism behind this life course regulation.

## Graphical abstract

The elegant work by Maejima et al. (Aging Cell, 2025) reveals a previously unrecognized mechanism linking age‐related oxytocin (OXT) decline to epigenetic remodeling, mitochondrial dysfunction, and systemic inflammation, reversible through exogenous OXT administration. This provides molecular evidence for the long‐proposed role of the OXT system as a regulator of health across the entire lifespan, from early development through aging, here, we extend this tantalizing glimpse into the epigenetic mechanism underlying life course regulation.

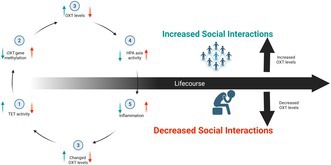

## 1 The Life Course Model: From Theory to Integrative Physiology and Molecular Mechanisms

Oxytocin (OXT) has been suggested to translate social experiences into physiological resilience and restoration in mammals (Moberg 2024, 2003; Uvanas‐Moberg et al. 2005; Uvnas‐Moberg 1998; Uvnas‐Moberg et al. 2024, 2014; Uvnas‐Moberg and Petersson 2005). Here, early closeness, care, and intimate social interaction act later in life to activate oxytocin neurons in the brain, stimulate social interactions, dampen hypothalamic–pituitary–adrenal (HPA) axis and sympathetic nervous system (SNS) activity, reduce inflammation, promote growth and restoration, enhance immune function, and support metabolic health. Our data showing that both endogenous oxytocin and exogenously administered oxytocin induce unexpectedly long‐lasting, sometimes life‐long stress reducing and growth promoting effects (Moberg 2024; Uvnas‐Moberg et al. 2024) has now received an explanation and a clear molecular mechanism. The Maejima findings demonstrate how OXT signaling actively maintains DNA demethylation through TET enzyme activity, enhancing and preserving the functional capacity of OXT neurons themselves, while simultaneously regulating stress levels, mitochondrial function, and inflammatory pathways (Maejima et al. 2025).

## 2 Early Neonatal Life and Development

Social experiences early in life may establish epigenetic patterns that govern OXT system function across decades. Birth, closeness, and maternal care stimulate sensory nerves through which the infant's central OXT system is activated, producing immediate and long‐term anti‐stress and growth‐promoting effects—the “calm and connection” system. Early closeness establishes enhanced OXT function that may persist into adulthood, and repeated administration of oxytocin postpartum may even reverse stress levels obtained in response to prenatal stress (Matthiesen et al. 2001; Stock and Uvnas‐Moberg 1988; Uvnas‐Moberg 2024; Uvnas‐Moberg et al. 2019, 2014; Uvnäs Moberg and Petersson 2022).

Maejima has now shown that this may operate through an epigenetic mechanism. Early‐life OXT activation likely establishes demethylation patterns in hypothalamic OXT neurons, setting a “baseline” level of OXT system function that the individual carries forward. The amount and quality of maternal care will influence the function of the OXT system. High levels will influence the OXT function in a positive way (Caldji et al. 2000), while early social deprivation or neglect will disrupt OXT signaling and may be shown to induce premature DNA hypermethylation of OXT neurons, accelerating the trajectory toward age‐related OXT decline (Bergman 2024).

## 3 Adulthood

The OXT system remains actively engaged by social interaction/closeness throughout adulthood, continuously modulating stress responses, cardiovascular function, and inflammatory tone. Social intimate relationships can now be conceptualized as physiological regulators of the OXT system resulting in the activation of the “calm and connection” system (Moberg 2024; Uvanas‐Moberg et al. 2005; Uvnas‐Moberg 1998).

The Maejima demonstration that OXT signaling maintains TET enzyme activity provides the complementary molecular basis of the long‐term active regulation. Each social interaction that stimulates OXT release may function as an “epigenetic maintenance event,” actively removing methyl groups that would otherwise accumulate on genes governing OXT production, mitochondrial function, and inflammatory control (Lu et al. 2015; Rasmussen and Helin 2016).

**FIGURE 1:**
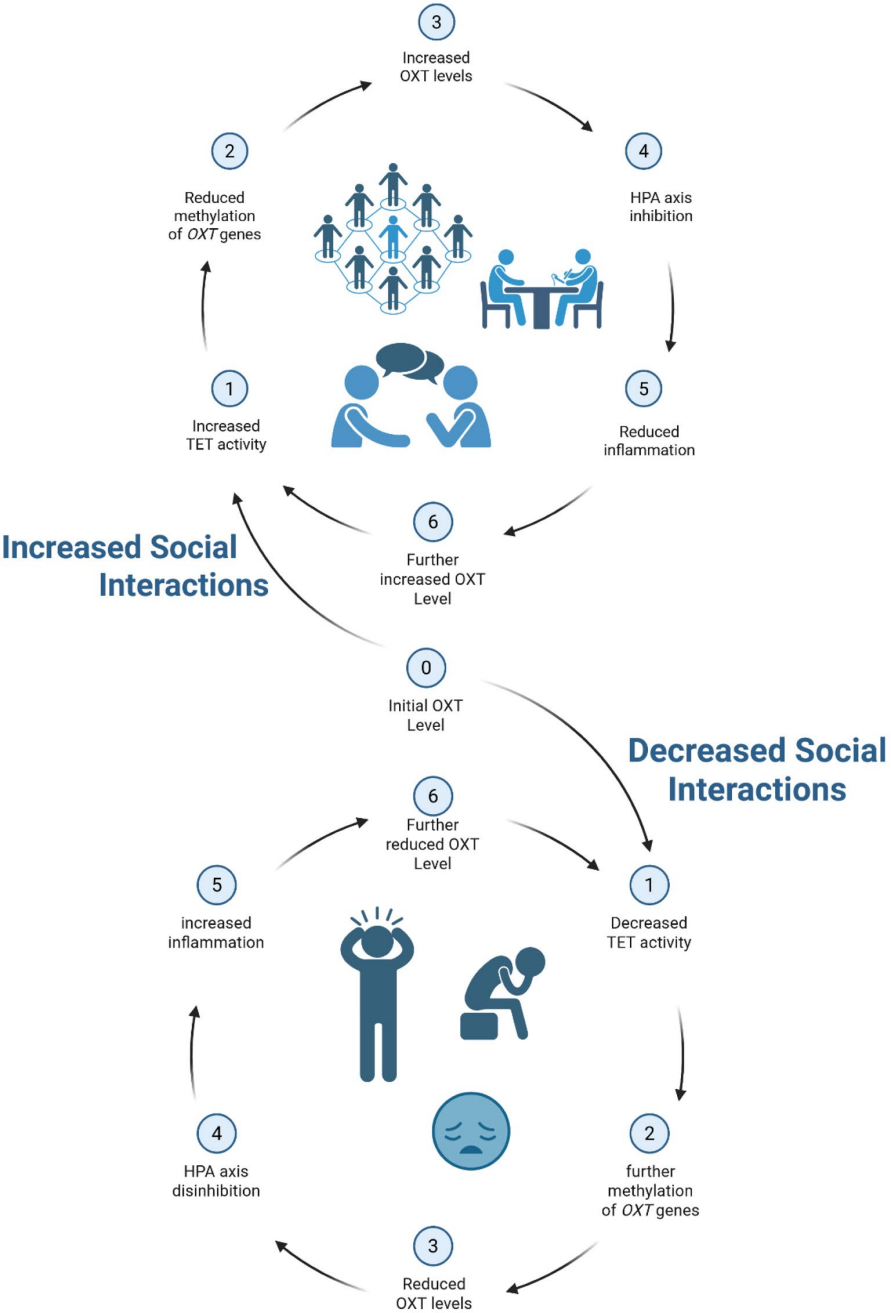
The Maejima molecular mechanism implies that OXT is both target and regulator of epigenetic state creating either a vicious or virtuous circle depending on social interactions and bonding. Aging or a lack of social contact silences *OXT* leading to less OXT, less TET2, more *OXT* methylation and further OXT suppression in a vicious cycle (lower loop). Inversely, their data suggest that OXT signaling could modulate the epigenetic tone of the HPA axis, perhaps even buffering or reversing epigenetic marks of chronic stress. Through social bonding, prosocial behavior (and possibly intranasal OXT) there is a form of “epigenetic maintenance event,” creating a virtuous circle (upper loop) whereby increased social bonding/interaction increases TET activity, reduces *OXT* methylation, further increasing OXT levels, inhibiting the HPA axis, reducing inflammation, which, in turn, further increases inflammation. In both cases, there is also a direct link from 3 to 5 by passing 4.

The Maejima data suggests that repeated activation of the OXT system by regular social engagement involving touch and intimate connection will maintain demethylated states through ongoing TET activity, creating a molecular “use it or lose it” loop where OXT neurons require regular activation to maintain their functional capacity (Uvnas‐Moberg et al. 2014; Uvnäs Moberg and Petersson 2022) (Figure 1). Inversely, prolonged social isolation reduces endogenous OXT stimulation, allowing or inducing progressive hypermethylation of OXT and related genes, in agreement with the known cardiovascular, restorative and immune consequences (Petersson et al. 1996); in other words, accelerated epigenetic aging of the OXT system.

Women who have breastfed and been exposed to high amounts of oxytocin are less likely to develop breast and ovarian cancer, as well as stress related diseases such as hypertension, diabetes, heart infarction, and stroke (Uvnas‐Moberg et al. 2019), suggesting an enhanced activity of the oxytocin mediated TET mediated demethylation process. Major life transitions in adulthood—partnering, parenting, divorce, bereavement—may also represent periods of rapid epigenetic remodeling (Aiello et al. 2024; Kim et al. 2024). Losses could accelerate methylation while positive intimate relationships restore it.

## 4 Late Life: Cumulative Effects and Intervention Opportunities

Older adults have diminished OXT levels, which may contribute to age‐related health vulnerability (Moberg 2003). Maejima et al. show progressive DNA hypermethylation of OXT neurons, creating a self‐perpetuating decline, as shown in Figure 1. This cycle parallels mechanisms described in DNA methylation aging clocks, where epigenetic drift accumulates progressively with age (Bell et al. 2019; Fraga et al. 2005). Critically, Maejima et al. show that 10 days of nasal OXT administration not only restored TET2 expression, 5hmC marks, and mitochondrial function, but plasma OXT levels also remained elevated after cessation of OXT administration, indicating an enhanced oxytocin neuron function.

## 5 Biological Aging Across the Lifespan: Stress—OXT Axis Balance

We have long‐proposed that the OXT system counter‐regulates the stress systems with the balance between the two determining stress resilience and health outcomes (Moberg 2024; Uvanas‐Moberg et al. 2005; Uvnas‐Moberg 1998; Uvnas‐Moberg et al. 2024). Maejima et al. provide molecular evidence for the epigenetic maintenance or erosion of this balance. During infancy, early OXT activation establishes demethylated states in both OXT neurons and potentially in glucocorticoid receptor genes in the hippocampus and prefrontal cortex and in alpha‐2 adrenoceptor genes in the locus coeruleus (LC), which by reducing noradrenergic tone decreases corticotropin releasing hormone (CRH), sets lifelong low stress reactivity (Caldji et al. 2000; Moberg 2024; Uvnas‐Moberg et al. 2024, 2014). Ongoing social intimate experiences will continue to shape this balance. Chronic stress or adversity may tip toward stress dominance and OXT methylation, while supportive relationships maintain OXT‐mediated buffering. Those with well‐maintained OXT systems (e.g., through high relationship quality) show rapid HPA and stress recovery; those with declining OXT show prolonged cortisol elevation, cardiovascular reactions, and inflammatory activation after stress (Neumann et al. 2000; Tops et al. 2007). Age‐related decline in OXT‐mediated inhibitory mechanisms contributes to HPA dysregulation and elevated basal cortisol levels as well as to hypertensive disorders observed in aging populations (Sapolsky et al. 2000).

## 6 Inflammation, a Common Currency

A key insight from both the physiological and molecular data is that positive intimate experiences decrease inflammation and biological aging. OXT exerts potent anti‐inflammatory effects throughout the body (Buemann et al. 2021; Clodi et al. 2008). Progressive OXT decline permits chronic low‐grade inflammaging, which then accelerates DNA methylation through multiple mechanisms—oxidative damage, altered metabolic cofactors (affecting TET enzyme function), and inflammatory signaling directly influencing DNA methyltransferases (Franceschi et al. 2018; Unnikrishnan et al. 2019). This relationship is bidirectional; TET2 loss in immune cells increases inflammation through enhanced IL‐1β production and NLRP3 inflammasome activation, contributing to age‐related cardiovascular disease and metabolic dysfunction (Fuster et al. 2020; Wang et al. 2024). If OXT maintains TET2 expression, then it may also protect against inflammaging through this epigenetic mechanism.

## 7 From Bench to Bedside: A Lifecourse Intervention Framework

The integration of our lifecourse model with the Maejima molecular mechanisms suggests a comprehensive approach to maintaining OXT system function across life stages (Table 1).

**TABLE 1:** The integration of our lifecourse model with the Maejima molecular mechanisms suggests a comprehensive approach to maintaining OXT system function across life stages.

Life stage	Epigenetic event	Functional effect	Health outcome
Early life	Maternal care shapes *OXT*, *OXTR* methylation	Increasing OXT cells and receptor function, resulting in reduced stress reactivity and increased restoration	Established social interactive behavior Reduced risk of stress related disease, growth
Adulthood	Chronic stress or social isolation maintains OXT suppression and high HPA/sympathetic nervous tone	Low oxytocin function high, stress levels, sustained inflammation, mitochondrial strain	Anxiety, depression, metabolic syndrome, CV disease
Aging	Hypothalamic *OXT* silencing via TET2/5hmC loss	Reduced OXT, less inhibition of CRH, chronic low‐grade cortisol and inflammation	Neurodegeneration, cognitive decline, frailty, further increased CV disease and metabolic syndrome
Intervention (exogenous OXT)	Nasal OXT reverses methylation of oxytocin and other neurons and mitochondrial deficits	Reestablishes oxytocin function, balances OXT/stress axis, lowers inflammation	Potential restoration of neural and cognitive function, stress resilience, reduced CV disease

Abbreviations: CV, cardiovascular; HPA, hypothalamus, pituitary, adrenal axis; OXT, oxytocin; OXTR, oxytocin receptor.

## 8 Conclusion: Social Connection as Lifelong Epigenetic Medicine

The work by Maejima et al. provides the first molecular validation of our lifecourse model of OXT as a master regulator translating social experience into physiological health. By revealing that OXT signaling actively maintains DNA demethylation through TET enzyme activity, we have a potential explanation of how lifelong relationship quality continuously shapes the epigenetic landscape governing stress resilience, inflammatory control, and biological aging rate.

This fundamentally reframes aging research. Intimate social relationships play a much bigger role in health maintenance than previously understood. Rather than viewing age‐related decline as inevitable cellular senescence, we can now understand it partly as accumulated epigenetic drift, for example, resulting from inadequate social‐physiological maintenance of the OXT system—a drift that appears reversible through restoration of OXT signaling.

The therapeutic implications are profound. Beyond pharmacological supplementation, this work provides molecular justification for public health initiatives supporting maternal–infant bonding, reducing childhood adversity, combating adult social isolation, and maintaining social connection in later life. These are not merely quality‐of‐life concerns but interventions operating at the most fundamental level of gene regulation, potentially slowing biological aging itself.

Perhaps most importantly, these findings affirm what has been long‐proposed: the important role of OXT during the entire life span. We are profoundly social creatures, and our relationships literally shape the pace at which we age. The OXT system, through its regulation of the epigenetic machinery governing its own production, functions as a molecular integrator of our social lives and our biology. Supporting this system across the lifecourse—from the first maternal touch to the last embrace—may be among the most powerful health‐promoting interventions available.

## Author Contributions

Kerstin Uvnäs‐Moberg, Jonathan D. Turner: conceptualization. All authors: literature review, manuscript writing, manuscript editing and read and approved the final version of the manuscript.

## Funding

The work of Jonathan D. Turner on the long‐term consequences of ELA was funded by FNR‐CORE (C20/BM/14766620 “ImmunoTwin”; C16/BM/11342695 “MetCOEPs”; C12/BM/3985792 “EpiPath”) and FNR‐INTER (INTER/ANR/16/11568350 “MADAM”). Kerstin Uvnäs‐Moberg, Jean Calleja‐Agius, and Jonathan D. Turner are Management Committee members, and MMG is a member of the EU funded COST action CA22114 “TREASURE” focused on early‐life and birth‐associated trauma.

## Conflicts of Interest

The authors declare no conflicts of interest.

## Data Availability

The authors have nothing to report.
